# An overview of select cannabis use and supply indicators pre- and post-legalization in Canada

**DOI:** 10.1186/s13011-021-00405-7

**Published:** 2021-10-07

**Authors:** Benedikt Fischer, Angelica Lee, Tessa Robinson, Wayne Hall

**Affiliations:** 1 Centre for Applied Research in Mental Health and Addiction (CARMHA), Faculty of Health Sciences, Simon Fraser University, Suite 2400 515 W. Hastings Street, Vancouver, British Columbia V6B 5K3 Canada; 2 Department of Psychiatry, University of Toronto, 250 College Street 8th floor, Toronto, Ontario M5T 1R8 Canada; 3 Department of Psychiatry, Federal University of São Paulo (UNIFESP), R. Dr. Ovídio Pires de Campos, São Paulo, 785 05403-90 Brazil; 4 Department of Health Research Methods, Evidence & Impact, Faculty of Health Sciences, McMaster University, Hamilton, ON Canada; 5 Centre for Youth Substance Abuse Research (CYSAR), 19 Upland Rd, St. Lucia, Queensland 4072 Australia; 6 National Addiction Centre, Institute of Psychiatry, Psychology and Neuroscience, King’s College London, 16 De Crespigny Park, London, SE5 8AF UK

**Keywords:** Canada, cannabis, Legalization, Outcomes, Population, Surveys, Use, Policy, Public health, Supply, Youth

## Abstract

**Background:**

Canada implemented the legalization and regulation of non-medical cannabis use, production and sale in 2018 aiming to improve public health and safety. While outcomes from legalization reforms in other jurisdictions mostly rely on US-based data have been assessed to be mixed, Canadian data are only emerging. We compiled select population-level data on key indicators to gauge initial developments from pre- to post-legalization of cannabis in Canada.

**Methods:**

We examined indicators data focusing on the following topics: prevalence of cannabis use, frequency of use, methods/products of consumption, driving after cannabis use, and cannabis sourcing. Indicator data were obtained mostly from national and some provincial population surveys. Prevalence or percentages for the indicators pre- and post-legalization (e.g., 2017- 2020), including confidence intervals were reported, with changes noted, as available in and indicated by the data sources.

**Results:**

Data suggested selected increases in cannabis use prevalence, mostly among mid- and older- but possibly also younger (e.g., under legal use age) users. Frequency of use and driving after cannabis use among active users do not appear to have changed. Methods of cannabis use show diversifying trends, with decreases in smoking and increases in alternatives use modes (e.g., edibles, vaping). There is a clearly increasing trend towards accessing cannabis from legal sources among adults, while under-legal-use-age youth do not appear to experience heightened barriers to obtaining cannabis in legalization contexts.

**Conclusions:**

Preliminary indicators on cannabis legalization in Canada show a mixed picture, some similar to US-based developments. While some use increases are observed, these do not necessarily represent indications of increases in cannabis-related harm, also since key (e.g., hospitalization or injury) data are lacking to date. There is a gradual embracing of legal supply sources of cannabis among users, which can be expected to serve public health and safety objectives. At the same time, cannabis use and access among under-age users as a principally vulnerable group do not appear to be hindered or reduced by legalization.

## Introduction

In October 2018, Canada implemented the legalization and regulation of non-medical cannabis use, production and sale. This policy was enacted to allow adults to use cannabis, reduce the illicit market, and improve public health and safety while minimizing cannabis use among youth [1, 2]. A handful of other jurisdictions have implemented non-medical cannabis legalization (e.g., Uruguay, several US states), are in the process of doing so (Mexico) or have considered it (e.g., Switzerland, New Zealand) [3].

Under Canada’s cannabis legalization regime, a mix of federal and provincial regulations stipulate a minimum age for legal cannabis use/purchase (18 years in Alberta, 21 in Quebec and 19 years in all other provinces), restrict places-of-use and ban advertising. They also regulate the legal availability of a variety of commercially produced cannabis (including herbal/flower, oils, extracts/concentrates, vape, edibles/drinkable) products via provincially managed (public and/or private) retail systems. There are provisions for limited ‘home-growing’ and a personal possession limit (30 g) for cannabis in most provinces. Cannabis-impaired driving is prohibited on the basis of defined per-se limits [4].

Cannabis legalization remains a ‘policy experiment’ with uncertain outcomes. The largest amount of evidence on the impacts of legalization has been based on US data that have shown mixed results [5, 6]. These include: some increases in cannabis use (mostly in older but also in other user groups) and use-related risk behaviors (e.g., increased frequency of use and the use of higher-potency cannabis products); a diversification of cannabis products and methods of use; and select increases in hospitalizations (e.g., for poisonings). There do not appear to be increases in treatment admissions for cannabis use disorders and data on changes in cannabis-related traffic fatalities are mixed [7–10]. Mixed results for key outcomes have been observed for Uruguay as well [11–13].

Canada represents an important case study for cannabis legalization. It is a G-7 nation that implemented a national, public health-oriented framework of legalization that may serve as a roadmap for other jurisdictions considering similar cannabis policy reforms. As argued elsewhere, the overall public health and safety impacts of cannabis legalization mostly depend on the development of a set of primary cannabis-related behaviors and harm outcomes [14]. As Canada finds itself approximately three-years since the implementation of non-medical cannabis legalization and in contexts of only limited other outcome data [15], we summarize selected key population-based indicators towards a preliminary assessment of developments in these outcomes from pre- to post- legalization of cannabis.

## Methods

We identified primary, population-based indicators of cannabis-related behaviors relevant to public health outcomes with pre- and post-legalization data. Where available, we used national, and some select provincial population data indicators, including from the following data sources. The *National Cannabis Survey (NCS) is* a provincially stratified, electronic household-survey (initial sample ~ 12,000 households) of a randomized sample of respondents aged 15 and up, which has been conducted in quarterly waves since 2018 to assess changes in cannabis-related behaviors and outcomes [16–18]. The *Canadian Cannabis Survey (CCS)* is an online survey that assesses cannabis-related behaviours and attitudes, based on an annual (since 2017), pan-Canadian, purposive, random-digit-dialing-recruited sample (> 10,000) of individuals aged 16 years and older [19–22]. *The Canadian Student Tobacco, Alcohol, and Drugs Survey (CSTADS)* is a biennial, stratified, pan-Canadian survey of secondary school students grades 7-12 (i.e., mostly ages 13-18 years) that collects data on substance use behaviors, attitudes and health [23–25]. *The Centre for Addiction and Mental Health (CAMH) Monitor* is a long-running, representative, telephone-recruited and -interview-based, biennial, cross-sectional household survey of the general adult population age 18 years+ in Ontario, Canada’s most populous province that focuses on substance use-related behaviors, attitudes and health [26]. The *International Cannabis Policy Study (ICPS)* is a multi-wave, web-based, consumer panel-based survey of a non-probability sample of respondents ages 16-65 conducted in Canada and select other countries. The survey’s wave 2 was conducted in Canada in September/October 2019 [1, 27]. The *Ontario Cannabis Store (OCS)* is a provincial agency that oversees and monitors legal cannabis distribution and retail operations in Ontario; in this capacity, it collects selected legal cannabis market data [28, 29]. We reported simple, sample-based prevalence or percentage values for the reported indicators for the total and select sub-populations for pre- and post-legalization data points as available. Corresponding further data details (e.g., 95% confidence intervals [95% CI]), and over-time changes are reported as indicated by the primary datasources’ information.

## Results

[for datasources utilized please refer to respective source references provided in the Methods].

### Prevalence of cannabis use

In the NCS, the total prevalence of cannabis use (in the past 3 months) increased from 14.9% (95% CI: 14.1, 15.7) in 2018 (Quarter [Q]1-3) to 16.8% (95% CI: 16.1, 17.6) in 2019 (Q1-4), to 20.0% (95% CI: 18.3, 21.8) in 2020 (Q4). The prevalence of use in each survey was respectively highest in those aged 18-24 years (30.9% [95% CI: 26.2, 36.1]; 33.3% [95% CI: 29.0, 37.8] and 35.6% [95% CI: 27.1, 45.1]). Among respondents aged 15-17 (i.e., those under the legal cannabis purchase age), use prevalence was 19.8% (95% CI: 13.4, 28.3) in 2018, 10.4% (95% CI: 6.9, 15.3) in 2019 and 19.2% (95% CI: 10.0, 33.6) in 2020; however, these estimates did not significantly differ due to small sample sizes.

Among CCS respondents, cannabis use (in the past 12 months) increased from 21.9% (95% CI: 21.1, 22.6) in 2018 to 24.6% (95% CI: 23.7, 25.4) in 2019 and 26.9% (95% CI: 26.0, 27.9) in 2020. Cannabis use was highest in the age group of 20-24 years (43.8% [95% CI: 40.3, 47.3], 51.3% [95% CI: 48.8, 53.8] and 52.5% [95% CI: 50.1, 54.9]) and also increased among those aged 16-19 years from 36.5% (95% CI:32.1, 41.1) in 2018, to 44.3% (95% CI: 40.7, 48.0) in 2019 and 43.5% (95% CI: 40.0, 47.1) in 2020.

In the CSTADS, cannabis use (in the past year) remained steady from 16.7% (95% CI: 15.5, 17.9) in 2016/17 to 18.1% (95% CI: 16.3, 19.8) in 2018/19. Cannabis use, however, increased among younger students in grades 7-9 (e.g., ages 13 – 15; from 5.5% [95% CI: 5.0, 6.1] in 2016/17 to 7.0% [95% CI: 6.2, 7.8] in 2018/19), while it remained unchanged in older students in grades 10-12 (27.8% [95% CI: 25.6, 29.9] in 2016/17 to 29.4% [95% CI: 26.2, 32.5] in 2018/19).

In the CAMH Monitor, the prevalence of cannabis use (in the past year) increased from 19.4% (95% CI: 17.3, 21.7) in 2017 to 25.6% (95% CI: 23.5, 27.7) in 2019. The prevalence of use was highest among 18–29-year-olds (39.1% [95% CI: 32.5, 46.1] to 45.5% [95% CI: 39.7, 51.4]). All age groups showed increasing trends in use between 2017 and 2019.

### Frequency of use

In the NCS, ‘daily/near-daily’ use of cannabis remained steady from 5.9% (95% CI: 5.4, 6.5) in 2018 (Q1-3) to 6.0% (95% CI: 5.5, 6.5) in 2019 (Q1-4) yet then increased to 7.9% (95% CI: 6.8, 9.2) in 2020 (Q4), based on the total respondent sample. On this basis, respectively corresponding estimates of the percentages of active cannabis users with daily/near daily use (e.g., 39.6, 35.7, 39.5%; these data not reported by the datasource) would be similar. In 2020 (Q4), the prevalence of daily/near-daily cannabis use in the total survey population was higher in those aged 18-24 years (16.3% [95% CI: 10.6, 24.3]) and in those aged 25-44 years (10.8% [95% CI: 8.5, 13.6]), than those 45 years and up (4.6% [95% CI: 3.8, 5.5) (respectively corresponding percentage estimates among active users would be 45.8, 35.6, 43.8%; these data not reported by the datasource).

In the CCS, the proportion of active cannabis users (past 12 months) who reported daily/near-daily use remained unchanged at: 24.9% (95% CI: 23.2, 26.7) in 2018, 23.9% (95% CI: 22.3, 25.5) in 2019 and 24.8% (95% CI: 23.2, 26.6) in 2020. In 2020, the rate of daily or almost daily use was highest among males (21.0% [95% CI: 18.9, 23.2]) and people aged 25+ years (18.4% [95% CI: 16.7, 20.3]).

In the CAMH Monitor, the rate of past-year cannabis users who endorsed a moderate or high-risk of cannabis use problems score on the Alcohol, Smoking, Substance Involvement Screening Test-Cannabis Involvement Score (ASSIST-CIS; which includes ‘frequency’ of use as one indicator) was measured at 53.3% (95% CI: 45.0, 61.3) in 2017 and 57.9% (95% CI: 51.7, 63.8) in 2019.

### Methods of consumption

In the NCS, ‘smoking’ remained the most common method of cannabis use (65.3% [95% CI: 60.6, 69.8] in Q1/2019 to 58.3% [95% CI: 53.3, 63.2] in Q4/2020), followed by consumption of ‘food or drink’ products (13.2% [95% CI: 10.1, 17.1] to 18.6% [95% CI: 14.8, 23.2]) and ‘vaping’ (12.9% [95% CI: 9.9, 16.6] to 11.9% [95% CI: 9.0, 15.4]). The correspondingly most common cannabis products consumed were ‘dried flower or leaf’ (79.2% [95% CI: 74.8, 83.0] in Q1/2018; 77.6% [95% CI: 73.9, 80.9] in Q1/2019; 70.9% [95% CI: 73.9, 80.9] in Q1/2020), ‘edibles’ (31.8% [95% CI: 27.1, 36.9], 29.1% [95% CI: 24.6, 34.0], 41.4% [95% CI: 36.5, 46.4]) and cannabis cartridges/vape pens (19.5% [95% CI: 15.3, 24.4], 18.4% [14.9, 22.6], 23.2% [95% CI: 19.3, 27.5]).

In the CCS, the prevalence of cannabis smoking decreased from 88.6% (95% CI: 87.3, 89.8) in 2018 to 84.0% (95% CI: 82.5, 85.4) in 2019 and 79.2% (95% CI: 77.6, 80.8) in 2020 but remained the overall most popular method of use. Conversely, ingesting cannabis products (e.g., as food/drink products) increased from 43% in 2018 to 48% in 2019 and 53% in 2020. Vaping cannabis (e.g., with a vape-pen or vaporizer) increased from 33% in 2018 to 36% in 2019, and decreased to 31% in 2020.

Among CSTADS’ Grade 7-12 students who used cannabis in the past year, ‘smoking’ cannabis (e.g., in a ‘joint’) was the most commonly reported method of use, although it decreased from 79.9% (95% CI: 78.6, 81.1) in 2016/17 to 76.4% (95% CI: 74.0, 78.8) in 2018/19. Conversely, the use of edibles increased from 34.2% (95% CI: 32.4, 36.1) to 45.4% (95% CI: 41.7, 49.2) alongside increases in the percentages of those reporting vaporizing/vaping (29.7% [95% CI: 27.6, 31.7] to 42.1% [95% CI: 38.5, 45.7]), and ‘dabbing’ (21.8% [95% CI: 20.4, 23.3] to 27.8% [95% CI: 25.5, 30.5]) in this period.

In the CAMH Monitor, smoking cannabis in a ‘joint’ (77.5% [95% CI: 70.22, 83.48] in 2017 to 79.2% [95% CI: 75.38, 21.21] in 2019), consuming cannabis as a food product (48.0% [95% CI: 39.96, 56.16] to 50.4% [95% CI: 45.60, 55.27]) and inhaling cannabis with a vaporizer [95% CI: 29.61, 42.12] to 32.6% [95% CI: 28.23, 37.29]) all remained steady as the most common modes of use among past-year users.

The OCS reported total sales of 30,000,000 g of legal cannabis products for Q3/2020, an increase of 16% over the previous quarter. The leading cannabis products by sales volume were ‘dried flower’ (57.4%), ‘vape’ products (15.7%), ‘pre-rolls’ (12.5%) and ‘edible’ products (4.5%).

### Driving after cannabis use

In the NCS, the prevalence of individuals who reported driving a vehicle within 2 h of cannabis consumption (in past 3 months) remained unchanged from 14.2% (95% CI: 12.1, 16.6) in Q1-Q3/2018 to 13.2% (95% CI: 11.4, 15.3) in Q1-4/2019.

In the CCS, the proportion of respondents reporting that they drove a vehicle after cannabis use in the past year (measured as within 2 h of any cannabis use in 2018, and 2 h after smoking/vaping and 4 h of ingesting a cannabis product in 2019 and 2020) decreased from 27% in 2018 to 24% in 2019 and 19% in 2020. This indicator appears to have decreased in each age sub-group.

In the CAMH Monitor, the proportion of adults in Ontario holding a driver’s license who drove a vehicle within 1 h of consuming cannabis (in the past year) was measured at 2.6% (95% CI: 1.7, 4.0) in 2017 and 3.1% (95% CI: 2.2, 4.3) in 2019. On assumption (made by the authors) that all persons who self-reported cannabis use also held a driver’s licence, these rates would translate to 13.4% (2017) and 12.1% (2019) of past-year cannabis users, respectively.

### Cannabis sourcing

In the NCS, the percentages of cannabis users who obtained cannabis from illegal sources (e.g., dealers) declined over time from 51.3% (95% CI: 46.1, 56.5) in Q1/2018 to 38.1% (95% CI: 33.7, 42.7) in Q1/2019 and 35.4% (95% CI: 30.6, 40.5) in Q1/2020. The same was the case for those sourcing their cannabis from ‘friends and family’ (47.0% (47.0% [95% CI: 41.8, 52.4], 37.0% [95% CI: 32.4, 41.9], 28.6% [95% CI: 24.0, 33.6]). Conversely, the proportions obtaining cannabis from legal sources increased (22.9% [95% CI: 18.8, 27.6], 47.4% [95% CI: 42.6, 52.2], 68.4% [95% CI: 63.8, 72.7]) as did those who grew their own or had it grown for them by someone else (8.0% [95% CI: 5.6, 11.3], 9.0% [95% CI: 6.4, 12.6], 14.2% [95% CI: 10.7, 18.6]). In 2020, the use of illegal sources remained highest among younger persons (ages 18-24 years; 56.6% [95% CI: 41.2, 70.8]) and legal sources were most commonly used by those aged 25-44 years (76.2% [95% CI: 70.4, 81.1]) and 45 years and older (68.1% [95% CI: 61.6, 74.0]).

In the CCS, the proportion of active cannabis users (past 12 months) who indicated that their usual source of cannabis was a ‘legal storefront’ increased from 24.5% (95% CI: 22.9, 26.1) in 2019 to 40.6% (95% CI: 38.7, 42.6) in 2020, while the percentage of those whose source was a ‘legal online source’ remained steady at 12.8% (95% CI: 11.5, 14.2) and 13.3% (95% CI: 12.0, 14.7), respectively.

In the ICPS, 47.7% of Canadian adult purchasers of dried cannabis flower products reported in fall 2019 that their last purchase was from a legal source, with, however, considerable inter-provincial variation (40.5–81.2%). The likelihood of purchasing a legal dried flower product was associated with distance (e.g., < 3 km; 1.56, 95% CI: 1.20, 2.02) and travel time (e.g., < 5 mins; 2.24 [1.56, 3.21]) to a legal outlet and with the frequency of cannabis use (*p* < .001 among daily/near-daily users compared to less-than-monthly users).

Among CSTADS’ secondary student respondents, who are below minimum age for cannabis use and purchasing under legalization, similar percentages (39% in 2016/17 to 40% in 2018/19) reported that cannabis would be “fairly easy” or “very easy” to obtain. The percentage who thought it would be “very or fairly difficult” to obtain cannabis decreased (46 to 42%). Moreover, in 2018/19, reflecting answers only from those respondents in the total sample who had previously obtained cannabis, about 4% indicated that it has been “easier”; less than 1% indicated it has been “harder” and 12% indicated that it has been “neither easier, nor harder” to obtain cannabis since legalization of cannabis use and sales for adults.

Based on cannabis market data from the OCS, the overall share of cannabis sales from legal sources in Ontario increased from 4.6% in Q4/2018 to 24.7% in Q4/2019 and 40.3% in Q3/2020.

## Discussion

The legalization of non-medical cannabis use in Canada occurred approximately three years ago. The Canadian population’s support of the policy has grown to a substantive majority (> 70%), but it is too early for a comprehensive assessment as to whether legalization in Canada has been a ‘successful’ policy experiment in achieving its public health and safety policy goals [2, 30]. Legalization requires time to be fully implemented and its effects to be assessed, but assessments to emerge are unlikely to be unequivocal or conclusive [5, 15, 31]. We provided an assessment of selected population-based cannabis use and supply indicators relevant to its public health and public safety outcomes from pre- to post-legalization. While the evolution of these indicators occurred over-time and is associated with legalization as a fundamental policy reform, the observations are mainly ecological in nature and possible changes do not allow for causal attribution to the intervention. Other, secular or independent factors may have driven or contributed to the developments observed.

On the basis of these data, we offer the following ‘big picture’ observations. First, the prevalence of cannabis use in the population may have slightly increased following legalization in Canada – a jurisdiction which already had a comparably high prevalence of use prior to legalization. The increases observed seem to have arisen primarily in mid- and older-age users but may also have occurred in those under the legal age of use, i.e., youth/teenagers whose access to cannabis was intended to be reduced by legalization. Such developments are confirmed by the international Tobacco Control Evaluation Project, where cannabis use (in the past 12 months) among respondents ages 16 to 19 years in Canada was found to have significantly increased between 2017 and 2019 (AOR 1.28, 95% CI 1.15–1.43; p<0.0001) [32]. Several of the increases in use also appear to extend pre-legalization trends. For example, in the CAMH Monitor, the increases observed in cannabis use for most age groups 2017 - 2019 extend pre-existing increase trends over multiple years prior to 2017. Moreover, the COVID-19 pandemic has been associated with increases in psychoactive substance use, including cannabis, that may have contributed to increases observed in 2020 data [33].

Second, there were no substantive signals of increases in daily- or near-daily use among current cannabis users, a primary predictor of increased risk of cannabis-related health harms (e.g., mental health and dependence) [34, 35]. However, these rates already have stood at comparably high levels on a population basis. Similarly, no discernable increases were noted in the population-prevalence of cannabis-impaired driving. Driving under the influence of cannabis is an important outcome indicator for public health because it increases the risk for motor-vehicle collisions and related injuries/deaths that represent a well-documented principal contributors to the cannabis-related burden of disease [36, 37]. This suggests that new deterrence-oriented legislation implemented with legalization and targeted prevention messaging in this area may have been effective [38, 39].

Third, there has been a steady diversification of modes of cannabis use and related cannabis products [40, 41]. While ‘smoking’ cannabis (e.g., ‘joints’) remains the predominant route of use among cannabis users in Canada, its overall share has been declining in favour of alternative use modes (e.g., vaping, edible use) and products (e.g., vape oils and edibles), all of which are legally supplied through the Canadian retail system established by legalization. Increases in these alternative use modes have also been reported by other Canadian study samples, including adolescent users [32]. These developments may lead to reductions in chronic health risks from cannabis smoking for users and others, while bringing possibly different challenges associated with alternative use modes (e.g., delayed effect onset and possible over-use/impairment from edibles, pulmonary health risks from vaping products) [42–44].

Fourth, there is evidence that Canadian cannabis users have been making a steady transition from ‘illegal’ to ‘legal’ sources of cannabis products. It is plausibly assumed that after 2020, the substantial majority of users will have been utilizing ‘legal’ sources to obtain their cannabis. As expected, it has been a gradual process for cannabis consumers to adjust to and embrace the availability of legal cannabis distribution after decades of forced reliance on the illegal cannabis market and supply [27, 45]. The percentage of users who access legal sources has also hinged on the gradual ramp-up and increasing availability of legal cannabis retail infrastructure and outlets, which has varied considerably across Canada [46, 47]. Increased legal cannabis access can be assumed to translate into ‘safer’ product use and related public health benefits based on the use of regulated, quality-controlled products [48]. On the other hand, the cannabis supply dynamics observed suggest that under-age cannabis users do not seem to experience increased difficulty with or reductions in access to cannabis products in the evolving reality contexts of legalization.

## Conclusions

The indicators reported are mostly drawn from population surveys based on self-report data, with limitations in sampling parameters, response rates and possible self-report biases, as well as inter-indicator differences in indicator specifics. Over the next years, additional data are expected to become available on key outcomes of cannabis legalization in Canada, adding to the evolving evidence base for assessing this ongoing policy reform experiment. Such assessments urgently require other essential indicators, including cannabis-related hospitalizations (e.g., for mental health problems), treatment admissions for cannabis use disorders and cannabis-related MVCs/injuries because these indicators represent principal drivers of cannabis-attributable burden of disease [14, 37, 49]. The preliminary data on cannabis legalization from Canada reflect some similar developments from the US, for example in regards to select increases in use and diversification in use methods yet an absence in clear change trends for driving under the influence of cannabis in legalization settings [7, 40, 50]. For now, currently available indicators suggest a ‘mixed picture’ of outcomes associated with legalization in Canada, including continuing high use rates of use among youth who are more vulnerable to adverse health effects of cannabis use than others and may be subject to punishments for ongoing use under legalization contexts [51, 52]. On this basis, policy efforts going forward should probably focus on measures and tools to reduce cannabis-related harms among under-age users, as well as reducing primary risk factors associated with adverse health outcomes among cannabis users generally (e.g., high-frequency use, risky use modes or products, cannabis-impaired driving, as furthermore defined in the ‘Lower-Risk Cannabis Use Guidelines’) mainly responsible for adverse public health impacts [53, 54].

## Data Availability

This manuscript reports on publicly available data.

## Abbreviations

US: United States
NCS: National Cannabis Survey
CCS: Canadian Cannabis Survey
CAMH: Centre for Addiction and Mental Health
CSTADS: Canadian Student Tobacco, Alcohol and Drug Survey
ICPS: The International Cannabis Policy Study
OCS: Ontario Cannabis Store
Q: Quarter
MVC: Motor Vehicle Collison
